# Engaging a Community in Developing an Entertainment–Education Spanish-Language Radio Novella Aimed at Reducing Chronic Disease Risk Factors, Alabama, 2010–2011

**DOI:** 10.5888/pcd9.110344

**Published:** 2012-08-02

**Authors:** Marcela Frazier, Shermetria Massingale, Michelle Bowen, Connie Kohler

**Affiliations:** Author Affiliations: Shermetria Massingale, Connie Kohler, University of Alabama at Birmingham, Birmingham, Alabama; Michelle Bowen, Media for Health, Birmingham, Alabama.

## Abstract

**Background:**

US Hispanics have disproportionate rates of diabetes and other chronic diseases. We used the entertainment–education approach to develop a Spanish-language radio novella aimed at reducing risk factors for diabetes, obesity, and tobacco use. The approach is based on social cognitive theory and proposes modeling as a source of vicarious learning of outcome and efficacy expectations.

**Community Context:**

The Hispanic population in Alabama increased by 145% between 2000 and 2010. Nearly one-quarter of Hispanics aged 18 to 64 live below the federal poverty level, and 49% are uninsured. Several lifestyle factors lead to poor health behaviors in this community. Radio is a popular medium among Hispanic immigrants. The single local Spanish-language radio station reaches a large proportion of the local community and several communities beyond.

**Methods:**

Through various methods, including workshops, review sessions, and other feedback mechanisms, we engaged stakeholders and community members in developing and evaluating a 48-episode radio novella to be broadcast as part of a variety show. We tracked participation of community members in all phases.

**Outcome:**

Community members participated significantly in developing, broadcasting, and evaluating the intervention. The desired outcome — development of a culturally relevant storyline that addresses salient health issues and resonates with the community — was realized.

**Interpretation:**

Our approach to community engagement can serve as a model for other organizations wishing to use community-based participatory methods in addressing Hispanic health issues. The radio novella was a unique approach for addressing health disparities among our community’s Hispanic population.

## Background

Hispanic adults in the United States have a 66% higher risk for diabetes than non-Hispanic white adults (1). From 2007 to 2009, 11.8% of Hispanic adults and 7.1% % of non-Hispanic white adults had diagnosed diabetes (1). Hispanic ethnicity has been identified as a diabetes risk factor (2). Diabetes prevention in this population is a national priority (3) for which various approaches exist (4,5). 

US Hispanics also have high rates of chronic liver disease, hypertension, heart disease, and obesity (6). The leading illnesses and causes of death among Hispanics are heart disease, cancer, unintentional injury, stroke, and diabetes (7). Physical activity levels are lower among Hispanics than among the general population (5,8).

We used the entertainment–education approach to develop a Spanish-language radio novella aimed at reducing risk factors for diabetes, obesity, and tobacco use. The approach is based on social cognitive theory that proposes modeling as a source of vicarious learning (9-11). Entertainment–education radio dramas have been effective in international settings (10-12) but have rarely been used in the United States (13,14).

Our process engaged community members in identifying knowledge, attitudes, and behaviors typical of the community and in telling stories about health issues based on personal experiences. We brought together community talent and professionals in writing and producing a 48-episode serial drama, *Promesas y Traiciones (Promises and Betrayals)*, that models preventive behaviors.

**Listen to 2 episodes of *Promesas y Traiciones***

Episode Four: *“La Carne es Débil” (“The Flesh is Weak”)*

A transcript of this episode is also available.

Episode Eleven: *“Malas Noticias” (“Bad News”)*

A transcript of this episode is also available.

**Podcast**

Listen to *PCD’*s audio podcast interview with the authors of this article as they describe how they used a unique entertainment–education model to develop a Spanish-language radio novella aimed at reducing the risk factors for chronic diseases among this population.

http://www2c.cdc.gov/podcasts/player.asp?f=8624562 (English)

http://www2c.cdc.gov/podcasts/player.asp?f=8624565 (Spanish)

## Community Context

The Hispanic population in Alabama grew by 145% between 2000 and 2010, to 185,609 people (15). In Alabama, 24% of Hispanics aged 18 to 64 live below the poverty level, 49% are uninsured (16), 8.1% reported being told they have diabetes, and another 2.6% reported pre- or borderline diabetes (17). The Hispanic population in the urban county in which we produced our program is approximately 25,680 (18), and most are Mexican. Radio is a popular medium for Hispanic immigrants, “. . . a valued and entrenched part of Hispanic consumers’ lives . . . a primary media platform for this ethnic group” (19). The single local Spanish-language radio station reaches 4 counties.

Our intervention included both overall health objectives and community engagement objectives. Health objectives were to increase knowledge of risk factors, symptoms, and preventive actions; increase perceived susceptibility to and perceived severity of diabetes; increase self-efficacy for physical activity and healthy eating; and increase self-reported physical activity and healthy eating behaviors. Intervention outcomes will be reported elsewhere after data are collected and analyzed.

Community engagement is a cornerstone of the entertainment–education approach. Community engagement includes activities that identify issues, provide real-life narratives for story development, and validate the authenticity of the dialogue. Audience perception that a program reflects their lives is important for adopting new behaviors (20). We identified 2 community groups: community residents and representatives from organizations in the local Hispanic community (stakeholders). Community engagement objectives were 1) to involve stakeholders in identifying health-related issues in the Hispanic community, providing assistance in identifying and involving community residents, providing guidance on resources for health services, and demonstrating support through general intervention endorsement; and 2) to involve community residents in developing, broadcasting, and evaluating the intervention. The desired community engagement outcome was a culturally relevant storyline that would address salient health issues and resonate with the local Hispanic community.

## Methods

### The intervention

The community-based participatory intervention aimed to develop, broadcast, and evaluate a radio drama that promotes healthy lifestyles in the Hispanic community. 

We developed 48 five-minute episodes of a serial radio drama that included romance, untimely death, secret affairs, and murder, with health issues carefully intertwined. The goal of the drama was to show, rather than tell about, consequences of certain behaviors and to increase listeners’ perceived self-efficacy by illustrating the roadblocks encountered and overcome by characters as they journeyed to change. Two 5-minute episodes were broadcast weekly as part of a longer show that had other health-related elements. These elements included expert interviews, listener calls, information about accessible venues for physical activity, and “Health Capsule” podcasts (www.cdc.gov/spanish/podcast.html#radio) produced by the Centers for Disease Control and Prevention (CDC). Listener participation was encouraged throughout the hour-long show. Community engagement began in March 2010 (Table 1).

**Table 1:** Timeline for Community Engagement Activities, Spanish-Language Radio Novella, Alabama, 2011

Timeline	Activity	Participants	Results
**2010**			
Mar	Coalition-building workshop	51 Stakeholders invited, 30 attended	Outline of health issues for values grid
Jun	Community creative workshop and story harvesting	25 Community members	Health issues and stories
Mar–Jun	Development of values grid	Stakeholders, community members, and authors	1 Values grid
Jun–Aug	Scriptwriter selection	11 Potential scriptwriters	1 Scriptwriter
Jun–Aug	Director selection	5 Potential directors	1 Director
Aug–Sep	Casting of actors	71 Actors auditioned	10 Actors
Sep–Nov	Development of plot lines and character map by scriptwriter	Scriptwriter and authors	Plot and characters
Oct–Jan 2011	Pilot-script development and scriptwriting of episodes 1–12	Scriptwriter and authors	12 Episodes
Oct–May 2011	Revision of scripts by technical advisor in health	Advisory group	48 Episodes
Dec–Feb 2011	Testing with members of target audience and revision of pilot scripts	60 Participants for pilot-episode listening sessions confirmed	49 Participants
**2011**			
Jan	Production of first 12 episodes	Entire creative team, sound engineer, director, project coordinator	12 Pilot episodes
Jan–May	Scriptwriting of episodes 13–48	Scriptwriter and authors	36 Episodes
Feb–Dec	Production and postproduction of episodes 13–48	Entire creative team, sound engineer, director, project coordinator	36 Episodes
Jun-Dec	Broadcast of two 5-min episodes per week	La Jefa Radio	48 Episodes

### Community engagement

With the help of the county health department, we identified and contacted representatives from institutions and organizations linked to the local Hispanic community, such as health and social service providers and Hispanic media. These representatives were asked to identify other potential stakeholders and to contact them on our behalf. The potential stakeholders included a community-based Hispanic service organization, a community-based Hispanic health organization, faculty of the University of Alabama at Birmingham (UAB), county health department officials, personnel from the Spanish-language radio station, and other community organizations. We invited 51 potential stakeholders to a coalition-building meeting at which we gave presentations about the local Hispanic health status and the entertainment–education approach to health promotion. We also discussed current issues affecting the local Hispanic community to be used in the initial development of the “values grid” (Table 2). The meeting concluded with recommendations for intervention development and commitments from the coalition to enlist community members.

**Table 2:** Values Grid for Issue of Obesity and Its Health Problems (Cardiovascular Disease, Diabetes, and Poor Nutrition), Developed With Community Input, Spanish-Language Radio Novella, Alabama, 2011

Description of Situation	Proposed Positive Values	Discouraged Negative Values
Sedentary lifestyle and lack of physical activity have become the norm for most Hispanics. In some cases, Hispanic people do not identify the available resources or opportunities for physical activities.	Hispanics and their families lead a lifestyle full of physical activity. Families and communities come up with opportunities for physical activities that are fun and entertaining — such as soccer and folkloric ballet for men and women. Families take advantage of local parks and school grounds to play and be active, and children are encouraged at school to participate in some physical activity daily.	Hispanics choose activities that require little physical activity, such as watching television, playing video games, and “hanging out,” which have health and economic consequences. Neighborhood playgrounds, parks, and school grounds are neglected and becoming dangerous.
Hispanic families often substitute healthy foods with more readily available but unhealthy foods. They often blame time limitations or a lack of access and resources.	Hispanics understand the benefits of eating (more energy, better performance, etc); they know how to read food labels and are aware of excess sodium, fat, and carbohydrates.	Families purchase prepared foods for convenience, and their diet is based on fast foods high in sugar and fat. Sugar-filled drinks are the norm.
Poor nutrition and consumption of food and drinks (sodas) high in fructose, sugars, and other carbohydrates are leading to a high prevalence of obesity and diabetes within the Hispanic community in Alabama.	Families prioritize healthy diets that use locally grown foods that are prepared in the home. People learn to identify healthy and affordable choices at local groceries, farmers markets, and local eateries.	Hispanics eat lots of fast foods and prepared foods containing simple carbohydrates and saturated fats. They prefer soda over water. Dietary habits lead to obesity, heart disease, and diabetes, which can cause strokes, blindness, and other disabilities and diseases.
Families don’t know the benefits of good nutrition and how to make healthy choices.	The Hispanic community is aware of the importance of good nutrition, and families incorporate nutritious foods such as vegetables and fruits into their diets. They limit the consumption of sodas.	Families don’t know the benefits of healthy eating or how to make good food choices, leading to diseases.

After the coalition-building meeting, we planned a workshop for community members and recruited participants through newspaper and radio announcements, word of mouth, and assistance from coalition partners. The workshop was conducted in Spanish and followed a model developed by PCI Media Impact, an entertainment–education organization with whom we partnered. The workshop began 1 evening with a fiesta-like atmosphere. On the second day of the workshop, we engaged participants in activities designed to elicit personal narratives on health issues (known as “story harvesting”). In 1 activity, we gave the participants written prompts on various health issues and asked them to write stories about these from personal experience or from stories they had heard in the community. For example, 1 participant wrote a story about how a friend had been diagnosed with diabetes after receiving the prompt “I hear about more and more people having diabetes all the time” (Box).

**Box. Example of a story written by a community participant at a workshop designed to elicit personal narratives on health issues.**

**Original Spanish version**

Era una vez una señora que vivía muy presionada con su trabajo, sus hijos y problemas familiares, por lo que no tenia tiempo para dedicarle a su salud o pensar un poco en ella; hasta que un día le empecé a ver un poco desmejorada y le comente que la notaba algo mal pero ella solo decía que eran los problemas que tenia con su marido. Hasta que decidió darse tiempo para checarse y le diagnosticaron diabetes. Mi amiga Carmen no lo podía creer porque decía que ella siempre había sido muy sana y estaba muy joven para padecer de esto. Por esa época el hermano de mi marido adelgazo mucho y estaba muy demacrado y le dijeron que era diabetes.

**Translated English version**

There was a lady who was under a lot of stress with her work, her children, and her family problems, that’s why she had no time to dedicate to her health or think a little about herself; until one day I started to notice a decline in her and I told her I noticed she was not looking well but she just said it was her problems with her husband. Until she decided to dedicate sometime to get checked and she was diagnosed with diabetes. My friend Carmen could not believe it because she would say she had always been so healthy and she was too young to have this ailment. Around the same time my husband’s brother lost a lot of weight and he was not looking well and they told him he had diabetes.

Another workshop activity was the further development of a “values grid,” the basis for framing the issues addressed in the drama (Table 2). The values grid is intended to be developed with input from people who reflect the community’s stance on issues. In another activity, participants were asked to dramatize their health issue of choice. Drama elements from this activity were used later, and participants were invited to audition for the cast.

We distributed an open call for scriptwriters nationally. Because we hoped to identify a local writer, we held an optional entertainment–education writing training session for local writers; we clarified that we would select a scriptwriter whose submission best satisfied our criteria, regardless of training attendance. We selected a native Spanish-speaking writer from another state who had entertainment–education writing experience and submitted the best sample scripts as judged by a panel of staff and community representatives. We provided the writer with the stories and health priorities that emerged from the workshops and delineated the health behavior objectives to incorporate into scripts. The writer submitted drafts, and we revised the episodes to ensure that health messages were appropriately included. An advisory group of native Spanish speakers reviewed the scripts for story quality and cultural relevance and provided feedback according to their areas of expertise. The advisory group consisted of a CDC diabetes educator, 2 radio station executives, a social services organization representative, and 2 community members. After a national search, we selected a director who is a Hispanic theater professor at a northeastern university. Acting auditions were held locally. Most actors were local residents; experience ranged from none to regular local productions. The final cast consisted of all but 1 actor from the immediate community (Figure). Production of the episodes took place at a local audio studio. Intervention team members listened to the episodes and requested revisions as necessary.

**Figure:**
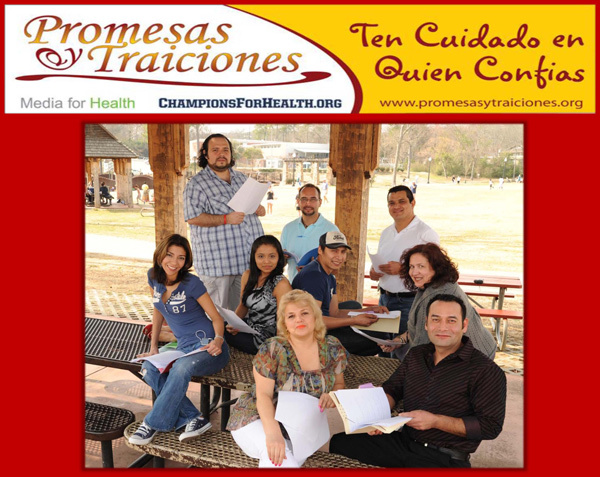
Cast of a 48-episode serial drama, *Promesas y Traiciones* (Promises and Betrayals), that modeled preventive health behaviors and was broadcast in 2011 through a Spanish-language radio station in Alabama.

A component of our formative phase was to test the potential efficacy of the first 12 episodes. This prebroadcast pilot testing included 4 listening sessions with a panel of Hispanic target audience members. Participants were recruited through advertisements in local Spanish-language newspapers, radio advertisements, and flyers. The listening sessions were approved by the UAB institutional review board, and each participant provided signed informed consent. We used a pretest–posttest questionnaire before the first listening session and after the fourth listening session. Measures were also pilot tested; they included knowledge of diabetes risk factors, symptoms, and preventive actions and measures of perceived susceptibility to and perceived severity of diabetes, self-efficacy for physical activity and healthy eating, and current behaviors. We constructed knowledge and attitude measures to directly reflect intervention objectives on the basis of a literature review and consultation with other diabetes prevention and control investigators. Behavioral measures were adapted from the Behavioral Risk Factors Surveillance System (17).

The questionnaire for the formative listening sessions was administered in Spanish. To minimize errors resulting from language and reading problems, we used an audience response system. Audience response systems allow audience members to respond to questions by pushing buttons on a remote-control “clicker.” Each question and its response options were projected in Spanish on a slide and read aloud by a local radio personality. The system records and stores each response immediately. An advantage of this system is that it decreases the potential for missing data; it monitors the number of participants who have responded and allows the investigator to encourage all of the participants to answer before advancing to the next question.

At the end of each session, we held a discussion on the content and entertainment value of the episodes, including such topics as cultural appropriateness (language, potential offensiveness, authenticity of characters and dialogue), impressions of what the characters are modeling, comprehension of embedded health messages, length, planned delivery format, likelihood of listening to the program, and likelihood of recommending it to others. We audio recorded the discussion and used it in further script development.

The 48-episode series was broadcast by the only local Spanish-language radio station. Two episodes were aired as part of a 1-hour variety show every Saturday for 24 weeks. The show included guest experts who responded to listener telephone calls. All calls made to the program were tracked and recorded. The number of calls served as an indicator of audience interest in the topic, and the content of calls provided a catalog of audience concerns.

We tracked planning, development, production, and formative evaluation activities. We collected quantitative and qualitative information, such as numbers of participants in each activity, outcome data from the listening sessions, and the kinds of questions asked during broadcasts. We summarized outcomes for each phase of the intervention.

## Outcome

Community members engaged in developing, broadcasting, and evaluating the intervention (Table 1). Thirty of 51 invitees participated in the coalition-building workshop, and 24 of 30 remained involved throughout the intervention in various capacities, such as community outreach, professional expertise, production, radio magazine guests, public relations support, and participation in community events.

Twenty-five community members participated in the community workshop; they identified various issues, including diabetes and health care access, and these were incorporated into the novella story line. The story line centered on an undocumented immigrant family, reflecting another prominent theme identified in the community workshop. Health issues were woven into the story line in a way that used characters to model the consequences of healthy and unhealthy behaviors and demonstrated the characters’ struggles to change to healthier behavior. All 48 episodes are available from www.promesasytraiciones.org.

The radio-show guest experts were primarily health professionals who discussed health and social topics, including diabetes, tobacco and drug use prevention, breast cancer, and physical activity. We received 92 listener telephone calls (Table 3).

**Table 3:** Sample Call-in Questions and Comments (N = 92) From Spanish-Language Radio Show Audience During 24 Weeks of Broadcast, Alabama, 2011

Topic
**Obesity (42 calls on such topics as prevention, diabetes, physical activity)**
My wife has been diagnosed with high cholesterol, but she refuses to do any exercise. I want to know if exercise will lower her cholesterol.
How many calories should an average person consume in a day?
I have 4 children, and during one of my pregnancies, I had gestational diabetes or something like that. My question is, I don’t have diabetes anymore. It was only during the pregnancy. In the future, am I going to have diabetes?
**Tobacco use (12 calls on such topics as prevention of addiction, second-hand smoke, nonsmokers’ rights)**
I clean the house of some Americans. What happens is that it is always smelling of cigarettes. They smoke a lot and have a lot of tobacco there. My question for her is can this affect me or my health? I almost never see them; they are always working. I have been working there a long time; the truth is that I almost never see them, but there is always that bad smell, and in the end I have to clean all of that.
Is there a way to impose a law that will prohibit parents from smoking around children? For example, I see parents smoking in the car with their kids, and they aren’t at fault that their parents smoke and that they are being exposed to this contaminant; they are innocent.
**Other (38 calls on such topics as domestic violence, breast cancer, intergenerational differences, and dental health)**
Communication is really important, and for that reason they look for other friendships. I have a son that is 22 years old. He got here when he was 7; he was really little. He made some bad friendships. He thought he was from here, and then he was deported. I want to give a message to parents: you need to take care of your children and stay on top of who they are hanging out with, what they are doing because since they think they are from here, and then later they get deported.
There are myths that a mammogram hurts a lot, that it gives you a lot of radiation. Are these myths true?

We updated stakeholders and community members through Constant Contact (Constant Contact, Inc, Waltham, Massachusetts) and regularly scheduled meetings. We submitted monthly reports to the county health department. The 2 local Spanish-language newspapers reported on the premier of the show (www.mediaforhealth.org/blog/news/2011/06/the-promesas-y-traiciones-in-latin-news/), and the radio station shared information about the program on the air and placed a link to our website on theirs (www.aquimandalajefa.com/index.html).

### Positive and negative aspects of the intervention

A positive aspect was the creation of community ownership through story harvesting in the community workshop; participants provided the creative team with relevant and powerful material for characters and plot. Community members continued to participate whether or not they became cast members. We created community excitement by posting calls for a director, scripts, and actors. Seventy-one Spanish-speaking community members auditioned for parts. We realized several long-term benefits. Creative partners (eg, writers, director, actors) gained a greater appreciation for community-based health-related work; we have had preliminary discussions with other entertainment–education producers to identify creative consultants for future work. We hope to continue our strong relationship with the Spanish-language radio station, whose owners have developed an appreciation for such community service activities as broadcasting health messages. We have broadened the network of organizations and individuals working with the Hispanic community. For example, members of our team were invited to work with Susan G. Komen for the Cure on an outreach program for Latinas, and others have joined UAB’s diabetes research group. The intervention was also publicized in the local English-language newspaper (http://blog.al.com/living-news/2011/09/local_radio_soap_operas_addres.html). Other positive aspects were the creation of a website, which allowed people to listen to the episodes outside of the radio coverage area; participation in local Latino festivals; use of social media to engage younger audiences and allow the actors to interact with audience members; and promotional items, such as water bottles and lunch bags, given to program callers.

The intervention also had several negative aspects. Initially, many community members did not trust the intervention partners, and we had difficulties in securing community participation. However, through a series of meetings and transparent processes, we reached a level of trust that enabled us to work effectively. Politics within the Hispanic community were challenging. For example, people who were accustomed to controlling community opinion withdrew their support when they disagreed with creative decisions. To address political issues, we maintained consistent, open communication. All community opinions were considered equally, and decisions were based on what we believed would best serve the community. We had limited staff resources and a small marketing budget. Marketing depended on word of mouth, the generous time given by the radio station, and a few strategically timed newspaper advertisements. Despite our small budget, as word circulated throughout the community, more people were willing to contribute to the production; contributions included additional grant money, volunteers, and discounted newspaper advertisements. New partners included both local Spanish-language newspapers, the Hugh Kaul Foundation, Susan G. Komen for the Cure, and locally owned Hispanic businesses.

A major unexpected challenge was the political atmosphere surrounding the immigration law passed by the Alabama legislature in summer 2011. The resulting community atmosphere of fear, uncertainty, and mistrust might have caused a decline in participation in traditionally well-attended community events. The local National Public Radio station produced a story about our intervention and the new immigration law (http://wbhm.org/News/2011/promisesandbetrayals.html).

## Interpretation

The benefits of the intervention on community engagement and ownership can serve as a model for other organizations attempting to use community-based participatory methods to address health issues in the Hispanic population.

An intervention like *Promesas y Traiciones* encompasses many different processes that must happen concurrently, and it must be designed to be flexible so that adjustments can be made easily. The processes are nonlinear and include participant recruitment, script-writer selection, advisory group enlistment, story and health message development, formative and process evaluation, director selection, acting-talent auditions, recording and postproduction, radio show development, public relations, and promotional-item selection. Ideally, planning should allow 24 to 36 months for such processes.

The production of a radio drama using the entertainment–education approach was a unique way to address health disparities among the local Hispanic population. It allowed for the inclusion of the community’s values, concerns, and perspectives. Stakeholders supported the intervention in critical areas of expertise and through general endorsement. Community members participated in all steps of the development, broadcast, and evaluation.

## Appendices

### Transcript for Episode 4 of *Promesas y Traiciones (Promises and Betrayals).*

This survey is available for download in Microsoft Word format. [DOC - 47 KB]

### Transcript for Episode 11 of *Promesas y Traiciones (Promises and Betrayals).*

This survey is available for download in Microsoft Word format. [DOC - 47 KB]
