# Supplementary material for: Engaging a Community in Developing an Entertainment–Education Spanish-Language Radio Novella Aimed at Reducing Chronic Disease Risk Factors, Alabama, 2010–2011
Source: Prev Chronic Dis. 2012 Aug 2;9:E134. doi: 10.5888/pcd9.110344 (PMC3475515; doi:10.5888/pcd9.110344)
Supplement: Supplementary file 3 [file 11_0344_e04.doc]

*Promesas y Traciones*

Episode 4 - Translation

“La Carne es Débil”

“The Flesh is Weak”

by Silvia Pontaza

Production Script

December 2010

In Order of Appearance:

Esteban

Rosita

Evelyn

Workers

Pancho

Mike

Voices

Office Johnson

MUSIC: SUSPENSE.

ESTEBAN

Un papel en blanco… pero ¿Qué podrá significar?

A blank piece of paper…but, what could it mean?

ROSITA

Pero si está en blanco, qué más puede ser sino una broma de mal

gusto.

If it is blank, what else could it be but a joke in bad taste?

SOUND: STEPS WALKING. DOOR SLAMS.

ROSITA

Ay Dios mío. Yo y mi bocota! Dime Evelyn, dije algo malo verdad?

Oh my God! Me and my big mouth! Tell me Evelyn, did I say something wrong?

EVELYN

Como si no conocieras a mi papá Rosita. Ya sabes lo que hace

cuando está pensando en algo…

As if you didn’t know my dad, Rosita. You know what he does when he needs to think…

ROSITA

Si. Es cierto. Ahora salió a fumar. Ya sé. (Suspira)

Yes, that is true. Now he has gone out to smoke. I know.(Sigh)

EVELYN

Y se va a estar allí afuera, preguntándole: “Cigarrillo, dime la

verdad?… dime cigarrito que piensas?” (ríe)

And he is going to be out there asking, “Cigarette, tell me the truth. Tell me Cigarette, what do you think?” (Laughs)

ROSITA

En mi pueblo había una señora que si le hablaba al cigarro. Y

luego, el cigarro le decía el futuro!

In my town, there was a woman who conversed with cigarettes and then later the ashes told her the future!

EVELYN

Ay Rosita no me digas que crees en esas cosas!

Oh! Rosita, don’t tell me that you believe in that stuff!

ROSITA

Bueno, yo no creo, pero tampoco dejo de creer! (Ríe)

Well, I don’t believe, but nor do I not believe! (Laughs)

SOUND: TRANSITION.

SOUND: AMBIENCE HOTEL KITCHEN.

WORKERS

Buenos días! Buenas Esteban.

Good Morning! Hey there Esteban.

PANCHO

Se apareció el Latin Lover!… Hola Esteban, ¿La pasaste bien

anoche?

The Latin Lover has appeared! Hey Esteban. Did you have a good time last night?

ESTEBAN

Ni me lo recuerdes… Ana se puso mal. Me quedé toda la noche en

la casa sin poder dormir.

Don’t even remind me! Ana was sick again. I stayed at home all night and couldn’t sleep a wink.

PANCHO

Tan mal estaba Ana?

Ana was that bad?

ESTEBAN

Ana? No, no fue por Ana. Me quedé despierto por otra cosa.

Cuéntame, ¿Cómo les fue ayer con la Migra?

Ana? No, it wasn’t because of Ana. I couldn’t sleep for another reason. Tell me, how did things go with immigration?

PANCHO

Se llevaron al colillas y a Laura, los demás estamos aquí.

They took Colillas and Laura. The rest of us are here.

ESTEBAN

Híjole, los van a deportar?

Wow! Are they going to deport them?

PANCHO

Pues quien sabe. El Bigotes dice que Bill se va a encargar.

Well, who knows! Bigotes says that Bill is going to take care of it.

ESTEBAN

De la que me salvé. Ay viene el Bigotes.

At least I was saved. Oh, look, here comes Bigotes.

SOUND: STEPS APPROACHING.

PANCHO

Bigotes… digo, Mike! Good morning!

Bogotes, Oh, I mean Mike! Good Morning!

MIKE

Buenos Días a todos. Estamos muy ocupados Si? Ayer dejamos cosas sin hacer y hay un banquete a las 11:00. Esteban encárgate.

Good Morning Everybody. We are very busy, right? Yesterday we left things undone and there is a banquet today at 11:00 am. You’re in charge, Esteban.

ESTEBAN

Si Mike. Todo va a estar listo a tiempo.

Yes, mike. Everything will be ready on time.

MIKE

Una cosa más. ¿Oficial?

One more thing. Officer?

SOUND: STEPS APPROACH, ECHOING.

VOICES

Ay no, otra vez.

Oh No! Not again!

MIKE

Muchachos, este es Oficial Johnson y está trabajando en una

investigación. Lo verán paseando por el hotel, por la cocina, no

tiene nada que ver con ustedes. ¿Verdad oficial?

Guys, this is Officer Johnson and he is working on an investigation. You will see him around the hotel and in the kitchen. It doesn’t have anything to do with you all. Right Officer?

JOHNSON

Correcto mundo. Solo sabe, yo voy a estar por aquí.

Correcto mundo. Just know that I will be around.

MIKE

Muy bien. Oficial, venga conmigo. Le mostrará todas las áreas.

Very good. Officer, come with me. I’ll show all the areas.

SOUND: STEPS OF MIKE AND OFFICER WALKING AWAY.

PANCHO

Lo que nos faltaba! Ahora resulta que nos van a estar vigilando.

Eso no lo pueden hacer!

Just what we needed! Now it appears that they are going to be watching us! They can’t do that!

ESTEBAN

Bueno a trabajar! Capulina, encárgate del caldo, Jo las cebollas y las ensalada y no muy salada porque protestan. Antonio, las

Charolas. Catracho, haz los desayunos, recuerda que a los

gabachos les gustan huevos con matequilla y sal para el

desayuno. Y para los que están a dieta solo los huevos, sin

mantequilla ni sal.

OK! Let’s get to work! Capulina, you take care of the soup. Jo, the onions and the salad, but not very salty because they will complain. Antonio, the trays. Catracho, make the breakfasts and remember that the Americans like eggs with butter and salt for breakfast. And those that are eating healthy, eggs without the butter and salt.

SOUND: SOUNDS OF KITCHEN. PLATES, FRYING PANS, CUTTING.

MUSIC: TRANSITION.

SOUND: AMBIENCE OF STREET. STEPS APPROACHING STOP SUDDENLY.

ROSITA

(Sorprendida) Evelyn! Qué estás haciendo afuera de la casa con

ese cigarrillo en la boca? Si tu papá te ve te mata… Nos mata!

(Surprised) Evelyn! What in the world are you doing outside the house with that cigarette in your mouth! If your father sees you he will kill you, he will kill us both!

EVELYN

Ay Rosita, no me regañes… estoy muy nerviosa!

Oh, Rosita. Don’t scold me. I am very nervous!

ROSITA

Pero no fumes. Eso es malo, estás muy joven para agarrar un

vicio así, después es más difícil dejarlo!

But don’t smoke! That is so bad. You are too young to start a habit like that and then it is even more difficult to quit!

EVELYN

Un cigarrillo me va a calmar los nervios. Además ¿Qué me puede

decir mi papá? Él también lo hace ¿Por qué no lo puedo hacer yo?

A cigarette will calm my nerves. Anyway, what can my dad say to me? If he does it, why can’t I?

ROSITA

¿Qué te pasa nena?

What’s wrong, Sweetie?

EVELYN

Hoy cuando salí de la escuela, alguien me venía siguiendo…

Today when I left school, someone was following me…

ROSITA

¿Alguien de la escuela? ¿Alguien que conoces?

Someone from school? Someone that you know?

EVELYN

No sé, solo sé que era un carro negro.

I don’t know- only that it was a black car.

ROSITA

A lo mejor fue tu imaginación… Si quieres voy por tí mañana.

It was probably your imagination. If you would like, I could pick you up tomorrow.

SOUND: CAR PASSING BY.

EVELYN

Mira! Ese se parece al carro que ví!

Look! That looks like the car I saw!

ROSITA

¿Ese que va allá?… También se parece al carro que yo vi

estacionado anoche aquí enfrente! ¿Estarán vigilando la casa?

That one right there? It also looks like the car I saw parked out front last night. Could someone be watching us?

MUSIC: SUSPENSE.

EVELYN

Tengo miedo Rosita!

I’m afraid, Rosita!

ROSITA

Vamos a entrar nena, no es seguro que estemos en la calle. Le

contaremos a tu papá cuando llegue del trabajo.

Let’s get inside, Sweetie. It is not safe for us to be out on the street. We will tell your dad when he gets home from work.

SOUND: DOOR OPENS AND CLOSES.

MUSIC: TRANSITION BACK TO THE KITCHEN. AMBIENCE OF CUTTING

BOARDS AND BOILING WATER, ETC. BACKGROUND ON THIS SCENE.

PANCHO

Entonces se te arruinó todo el pastel (Ríe) ¿Ves hermano? Por

algo pasan las cosas… A lo mejor no debes andar con esa vieja.

So, your cake was totally ruined last night? (Laughs) See brother? Everything happens for a reason. It is probably better that you don’t hang out with that old lady.

ESTEBAN

No sé Pancho, a veces me pongo a pensar en que no debo, pero ella me sigue llamando, y la carne es débil…

I don’t know Pancho. Sometimes I get to thinking that I shouldn’t, but she keeps calling me, and the flesh is weak…

PANCHO

Esa señora MacLana va a traerte problemas… la gente con dinero

siempre se sale con la suya.

That lady, Miss Money-Bags, is going to bring you nothing but trouble. People with money always end up on top.

ESTEBAN

Por el otro lado, me siento mal por Ana.

On the other hand, I feel bad for Ana.

PANCHO

¿Qué es lo que le pasa a Ana?

Now what has happened with Ana?

ESTEBAN

Desde que le diagnosticaron diabetes le molestan las piernas,

los ojos, tiene un sin fin de cosas que para que te cuento…

Since they diagnosed her with diabetes, her legs bother her, her eyes, she has an endless list of things wrong with her...there is no point going into it.

PANCHO

¿Y eso qué? Mucha gente vive con diabetes toda su vida!

¿Recuerdas que eso es lo que padece el tío Beto?

And what is up with that? Lots of people live with diabetes all their lives! Remember, Uncle Beto has that, too.

ESTEBAN

Si pero tío Beto se cuida, hace ejercicio y come saludable, pero

Ana no coopera! Nunca obedece al doctor! Es más, se le ha metido

en la cabeza que ya se va morir. Anoche me hizo jurarle que

cuando se muera voy a poner a Evelyn en la universidad…

Yes, but Uncle Beto takes care of himself. He exercises and eats healthy. Ana refuses to cooperate! She never does what the doctor tells her! And on top of that, she has gotten it in her head that she is going to die. Last night she made me swear that when she dies that I will make possible for Evelyn to attend college.

PANCHO

¿Y qué tal si no se muere nunca?

And what if she never dies?

ESTEBAN

Cállate baboso, contigo no se puede hablar en serio.

Shut up idiot. With you, no one can talk seriously.

SOUND: CELLPHONE RINGS SEVERAL TIMES, PANCHO SPEAKS AND THE

CELLPHONE CONTINUES RINGING IN THE BACKGROUND. THEN EVENTUALLY

DIES.

PANCHO

No vas a contestar? (pausa) Es Concha Macbilletes verdad?

(pausa) Mira! Qué honesto me saliste!(pausa) Ya te arrepentiste

de ponerle los cuernos a tu mujer! (Pausa) Eso! Que fuerza de

voluntad! Qué lástima porque la Concha esa está como quiere.

You are not going to answer? (pause) It’s Miss Concha McDollars, isn’t it? (pause) Look! How noble you are becoming! (pause) Now you are regretting pulling the wool over your wife’s eyes! (Pause) That’s it! What amazing self-control! What a shame because that Concha is a hot babe.

SOUND: CELLPHONE STARTS RINGING AGAIN. IT STOPS WHEN ESTEBAN

ANSWERS. PHONE CLICKS.

ESTEBAN

(Suspira) ¿Hola Concha? Si soy yo Esteban.

(Sigh) Hi, Concha? It’s me, Esteban.

END EPISODE 4.
