# Supplementary material for: Engaging a Community in Developing an Entertainment–Education Spanish-Language Radio Novella Aimed at Reducing Chronic Disease Risk Factors, Alabama, 2010–2011
Source: Prev Chronic Dis. 2012 Aug 2;9:E134. doi: 10.5888/pcd9.110344 (PMC3475515; doi:10.5888/pcd9.110344)
Supplement: Supplementary file 4 [file 11_0344_e11.doc]

*Promesas y Traiciones*

Episode 11 - Translations

“Malas Noticias”

“Bad News”

by Silvia Pontaza

Production script

December 2010

In Order of Appearance:

Esteban

Evelyn

Concha

Pancho

Doctor

SOUND: HOSPITAL AMBIENCE.

ESTEBAN

Evelyn, ya es tarde, mañana tienes que ir a la escuela, te tienes que ir a la casa.

Evelyn, it is late, and tomorrow you have to go to school. You have to go on home.

EVELYN

No yo me quiero quedar aquí en el hospital. El doctor no nos ha dicho como sigue mi mamá…

No, I want to stay here at the hospital. The doctor hasn’t told us how mom is doing.

ESTEBAN

No puedes esperar aquí toda la noche, vamos, te voy a llevar a la casa.

You can’t wait here all night. Come on let’s go. I am going to take you to the house.

EVELYN

No puedes llevarme, tú no tienes el coche aquí. ¿Recuerdas?

You can’t take me. You don’t have the car here, remember?

ESTEBAN

Ah, es cierto, mi coche se quedó en el hotel… Entonces voy a llamar al Pancho para que te venga a buscar.

Oh, that is true. My car is at the hotel. Then I am going to call Pancho to come and get you.

EVELYN

¡Ay no, el tío Pancho no! ¿Por qué me quieres torturar?

Oh! No! Uncle Pancho? No! Why do you want to torture me?

ESTEBAN

No tenemos de otra. Voy a llamarlo para que venga por ti, te lleve a la casa, y se acabó.

We don’t have a choice. I am going to call him to come and get you and take you home and that is the way it is going to be!

SOUND: DIALING NUMBER. CALLING SIGNAL. CELLPHONE RINGS SEVERAL TIMES.

CONCHA

¿No piensas contestar ese teléfono Pancho?

You aren’t going to answer the phone Pancho?

PANCHO

No. Ya vi que es Esteban.

No. I already saw that it is Esteban.

CONCHA

(Ríe) Ahora si te metiste en un lío con tu hermano.

(Laugh) Now you really got yourself into a mess with your brother.

PANCHO

No. No lo creo Concha.

No. No, I don’t think so, Concha.

CONCHA

Se supone que solamente venías a darme un recado de parte de Esteban, y terminaste acostándote conmigo, animal…

You were only supposed to come to give me a message from Esteban and you ended up sleeping with me, you animal…

PANCHO

Si, soy un animal, para todo.

Yes, I am an animal, at everything.

CONCHA

No respetas ni a tu propio hermano.

You don’t even respect your own brother.

PANCHO

Te recuerdo que somos MEDIO hermanos… aunque su mamá nos haya criado a los dos… yo vengo de otro lugar.

I will remind you that we are HALF brothers, and although his mother raised us both, I come from a different place.

CONCHA

¿Y por eso eres capaz de todo?

And because of that, you are capable of anything?

PANCHO

Reina, como él siempre tuvo más que yo… yo siempre tuve que ganármelo todo a pulso.

My queen, since he always had more than me, I always had to take things as I could get them.

CONCHA

¿Ganártelo a pulso? Yo más bien veo a un mocoso que quiere el helado del otro…

Take things as you could get them? I think it might be more like a little rascal that wants someone else’s ice cream.

PANCHO

¿Cómo? ¿Te refieres a ti? (Ríe)

What? You’re referring to yourself? (Laugh)

CONCHA

¡Claro! ¿Quién más? (Ríe) ¿Qué hora es? Ah… ¿Me pasas la bolsita que está sobre la mesa? Si, esa que está allí… gracias… Te diré una cosa corazón… yo al que quiero es a Esteban.

Of course, who else? (Laugh) What time is it? Oh! Can you pass me the bag on top of the table? Yes, that one there. Thanks. I will tell you something, Sweetheart. The man I love is Esteban.

PANCHO

El mismo Esteban ha dicho que tú eres una aventura nada más… ¿Qué haces?

Esteban himself has said that you are nothing more than a fling. What are you doing?

CONCHA

Me estoy picando el dedo ¿Qué no ves?

I am pricking my finger. Can’t you see?

PANCHO

¿Para qué?

What for?

CONCHA

Para medirme el azúcar en la sangre.

To measure my blood sugar.

PANCHO

¿Tienes diabetis?

You have diabetis?

CONCHA

No se dice diabetis. Y sí, tengo diabetes.

You don’t say diabetis. And yes, I have diabetes.

PANCHO

¿Y haces eso a cada rato? Yo nunca podría picarme así.

And you do that every so often? I could never prick myself like that.

CONCHA

Por lo menos dos veces al día, ya me acostumbré. Tengo que tomar medicamentos y llevar una dieta sana, también hago ejercicio…

At least twice a day, but I am used to it. I have to take medicine and follow a healthy diet. I also exercise.

PANCHO

Si, se ve que haces mucho ejercicio, estás hermosa. Oye, pero yo pensaba que esa enfermedad era solo de gordos…

Yes, I can tell you do a lot of exercise. You are beautiful. Hey, but I thought that was an illness that only fat people get…

CONCHA

Tiene mucho que ver lo que comes, no necesariamente tienes que ser gordo. Como Latinos, tenemos predisposición genética.

It has a lot to do with what you eat. You don’t necessarily have to be fat. As Latinos, we have a genetic predisposition.

PANCHO

Eso es lo que tiene Ana, y se está muriendo.

That is what Ana has, and she is dying.

CONCHA

Por supuesto que se puede complicar si no me cuido, yo he tenido diabetes desde hace unos cuantos años… y aquí me ves.

Of course I could have complications if I didn’t take care of myself. I have had diabetes for some time, and here I am!

PANCHO

Ya entendí… a Esteban le gustan las diabéticas (Ríe)

Now I get the picture… Esteban loves diabetics! (Laugh)

CONCHA

No seas tonto. Esto no es un juego, es una enfermedad muy seria que no tiene cura. Aquí está tu ropa. Ya te tienes que ir.

Don’t be stupid. This is not a joke. It is a very serious illness that doesn’t have a cure. Here are your clothes. Now it is time for you to leave.

PANCHO

Entonces qué… ¿Vas a andar con los dos, o que?

So, now what? Are you are going to continue on with both of us, or what?

CONCHA

¿Qué?! ¡Estás loco de remate! Esteban es mi hombre.

What? Are you a crazy lunatic? Esteban is my man.

PANCHO

Esteban se va a enterar de esto, y se acabó lo de ustedes.

Esteban is going to find out about this, and then you two will be over.

CONCHA

Antes de que abras esa boquita yo le estaré diciendo que te aprovechaste de mi… a ver como se lo explicas a la policía.

Te entraste a mi casa cuando yo estaba durmiendo, hay rastros de ti abriendo la puerta ¡Te tengo hasta en video! Yo nunca te he llamado en mi vida…

Before you open that little mouth of yours, I will be telling him that you took advantage of me. Let’s see, how you would explain it to the police? You entered my house while I was asleep. There is evidence of you opening the door- I have you on video! I have never even called you in my life…

PANCHO

Sabes jugar tus cartas… eso me gusta.

You know how to play your cards. I like that.

CONCHA

Como no tienes idea. Es más, sería gracioso… ¿Por qué no lo intentas?

You have no idea. Anyway, it would be funny. Why not try it?

PANCHO

No, ya obtuve lo que quería. Le gané a Esteban esta vez.

No, I got what I wanted. I one-upped Esteban once again.

CONCHA

A Esteban nunca podrás ganarle… Ten. El resto te lo pones afuera… ya no quiero que estés aquí, adiós.

You could never one-up Esteban. Take this, you can finish getting dressed outside. I don’t want you here anymore. Good Bye.

SOUND: DOOR IS SLAMMED.

PANCHO

De mi nadie se burla.

Nobody makes fun of me.

SOUND: CELLPHONE RINGS. CLICKS.

PANCHO

(Enojado) ¡Aló! Si soy yo Esteban… No no me había dormido, estoy todavía en la calle haciendo unos mandados… (Pausa) Todavía tengo la troca del hotel conmigo. (Pausa) Está bien, yo paso por ella al hospital. Adiós.

(Angry) Hello! Yes, it’s me, Esteban. No, I didn’t fall asleep, I am still out doing errands.. (Pause) I still have the hotel truck with me. OK, I will stop by the hospital to pick her up. Bye.

SOUND: CELLPHONE CLICKS

SOUND: HOSPITAL AMBIENCE.

ESTEBAN

Listo mija, Pancho va a ir a dejar la troca al hotel y luego te viene a buscar.

It’s all arranged, daughter, Pancho is going to leave the truck at the hotel and then he will be by to get you.

EVELYN

Prefiero quedarme toda la noche sin pegar un ojo, que irme en el coche con el tío Pancho…

I prefer to stay here all night without sleeping a wink than to get in the car with Uncle Pancho.

ESTEBAN

Ay Evelyn no exageres. Sea como sea nos está haciendo un favor. Pancho siempre está allí cuando lo necesitamos. Es familia.

Oh, Evelyn, don’t exaggerate. Be that as it may, he is doing us a favor. Your Uncle is always there when we need him. He is family.

EVELYN

Nunca lo he tragado… Papá mira allí está el doctor… ¿Viene para acá?

I have never been able to stand him… Dad look, there is the doctor. Is he coming over here?

SOUND: STEPS APPROACHING.

ESTEBAN

Parece que sí.

It would seem so.

DOCTOR

¿Señor Vidal?

Mr. Vidal?

ESTEBAN

Si doctor ¿Qué noticias nos tiene?

Yes, doctor. What news do you have for us?

DOCTOR

¿Puedo hablar con usted a solas?

May I speak with you alone?

EVELYN

Conmigo también… yo soy la hij…

You can talk to me, too. I am the daug…

ESTEBAN

Evelyn. Espérame aquí, voy con el doctor ya vuelvo.

Evelyn, wait for me here. I’m going with the doctor and I will be right back.

EVELYN

¡Pero papá…!

But Dad…!!

SOUND: STEPS WALKING.

DOCTOR

Perdone que pedí hablar con usted únicamente, no sé como quiere manejar con su familia la noticia que le voy a dar…

Pardon that I asked to speak with you alone. I don’t know how you want to handle the news I am about to give you with your family….

ESTEBAN

No se preocupe doctor, así está bien. ¿Qué es lo que pasa?

Don’t worry, Doctor. This is fine. What is going on?

DOCTOR

Lo que sucede es que, como le comenté anteriormente, la condición de su esposa se prestaba para muchas otras complicaciones… señor Vidal, su esposa Ana falleció.

What has happened is that… as I was telling you earlier, your wife’s condition could lead to many other complications- Mr. Vidal, your wife has passed away.

MUSIC: SUSPENSE.

END EPISODE 11.
